# All Dielectric Transmissive Structural Multicolor Pixel Incorporating a Resonant Grating in Hydrogenated Amorphous Silicon

**DOI:** 10.1038/s41598-017-14093-6

**Published:** 2017-10-19

**Authors:** Ishwor Koirala, Vivek Raj Shrestha, Chul-Soon Park, Song Gao, Sang-Shin Lee, Duk-Yong Choi

**Affiliations:** 1 Department of Electronic Engineering, Kwangwoon University, 20 Kwangwoon-ro, Nowon-gu, Seoul, 01897 South Korea; 2 School of Physics, The University of Melbourne, Melbourne, Victoria 3010 Australia; 3 Laser Physics Centre, Research School of Physics and Engineering, Australian National University, Canberra, ACT 2601 Australia

## Abstract

All dielectric transmissive type polarization-tuned structural multicolor pixels (MCPs) are proposed and demonstrated based on a one-dimensional hydrogenated amorphous silicon (a-Si:H) grating integrated with a silicon nitride waveguide. Both bandpass and bandstop transmission filtering characteristics in the visible regime, centered at the same wavelength, have been achieved by tailoring the structural parameters including the duty ratio of the grating and the thickness of the dielectric waveguide. For the three manufactured MCPs, the transmission peak exceeds 70% for the transverse electric (TE) polarization and 90% for the transverse magnetic (TM) polarization as observed at the resonance and off-resonance wavelength, respectively. The polarization-switched transmissions are attributed to the guided mode resonance initiated by the interaction of the a-Si:H grating and the dielectric waveguide. A broad color palette covering the entire visible band was successfully realized from a suite of MCPs with varying grating pitches. The proposed structural color pixels are expected to facilitate the construction of dynamic displays, image sensors, optical data storage, security tags, and so forth.

## Introduction

Color filtering pixels have been widely used in a variety of applications, multicolor imaging devices, dynamic displays, image sensors, biosensors, plastic consumer products, and visible light communications^1–6^. In particular, nano-structural color pixels have attracted immense attention in comparison with pigment-based colorants due to their high resolution, low photodegradation, cost effectiveness, stable colors, and recyclable materials^1–3^. The transfer characteristics for the plasmonic color filtering devices based on metals^6–8^, may need to be ameliorated in terms of the spectral shape, bandwidth, and efficiency. Silicon, which is the second most abundant element in the Earth’s crust, can be utilized for the large-scale fabrication of functional devices through the well-established and prevailing complementary metal-oxide-semiconductor (CMOS) process^9–16^. It is noted that hydrogenated amorphous silicon (a-Si:H) can be exploited to produce highly efficient, eco-friendly color pixels^14–16^, whose mechanism is based on guided mode resonance (GMR)^14^, allowing for large-scale deposition at low temperature on substrates like glass, dielectric, plastic, and metal. a-Si:H is known to be nearly impervious to oxidation, as opposed to amorphous silicon (a-Si), which is vulnerable to oxidation due to the free dangling bonds^16^. Several color pixels resorting to a cylindrical structure or two-dimensional (2D) grating in a-Si were previously reported^12,13^, they are mostly limited to a static case where only a single color is generated. For the realization of the controllable color output leading to compact and highly advanced color pixels^17–19^ devices and applications, various polarization-dependent color filter had been realized based on the complex structure including metal that is supported by plasmonic^20–23^ but with lower efficiencies. Grating based GMR color pixels are especially attractive for their compact design and fabrication and well-known to perform flexible power transfer characteristics with proper bandwidth and color purity^10,14,17^. Although there have been a few reports on all-dielectric GMR color pixels based on poly-silicon and crystalline silicon in transmissive mode^9,18,19^ but these are limited to static coloration. It is noted that from the viewpoint of the optical loss, a-Si:H is deemed to be comparable to polycrystalline silicon, which is obtained by thermally annealing a-Si. The all-dielectric grating based polarization-controlled color pixel has been demonstrated in reflection mode^17^ while an all-dielectric transmissive tunable color pixel lacks its investigation. Here, we report an all-dielectric polarization-tuned transmissive color pixel which capitalizes 1D a-Si:H based on GMR for the first time.

In this work, we propose and demonstrate an all dielectric polarization-tailored structural multicolor pixels (MCPs) based on an a-Si:H resonant grating, which are capable of responding to the transverse electric (TE) and transverse magnetic (TM) polarizations, so as to render vivid colors in additive-RGB and subtractive-CMY mode corresponding to the transmission peak and dip, respectively. The transmission peak and dip, occurring at the same wavelength, are confirmed to stem from the GMR via the inspection of the near-field intensity profile; meanwhile, the theoretical GMR conditions categorically correlate well with the simulated transmission characteristics. The a-Si:H resonant grating has been thoroughly studied from the standpoint of color tuning by adjusting the periodicity of the grating and the polarization angle, thereby facilitating the creation of a broad range of color images. The proposed structural MCP, which is notable for its capacity to exhibit enhanced angular tolerance, is anticipated to play a vital role in polarization-sensitive security tags, anti-counterfeiting, holographic devices, ultrafast display devices, and optical data storage^4,5,20–24^.

## Results

### Polarization-tuned transmission spectra of the proposed MCP and its color images

**Figure 1:**
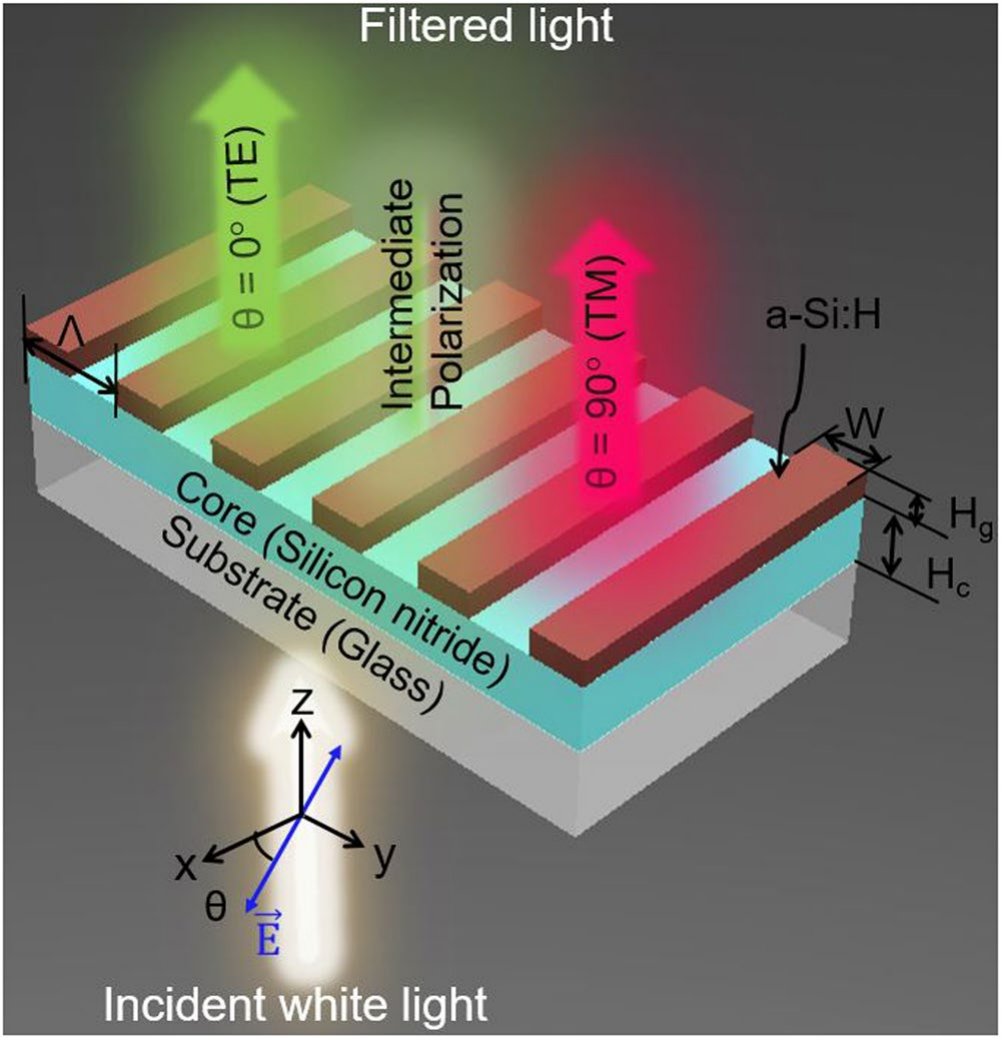
Configuration of the proposed structural multicolor pixel capitalizing on an a-Si:H resonant grating. Incident white light is filtered into different colors in accordance with the polarization.

**Figure 2:**
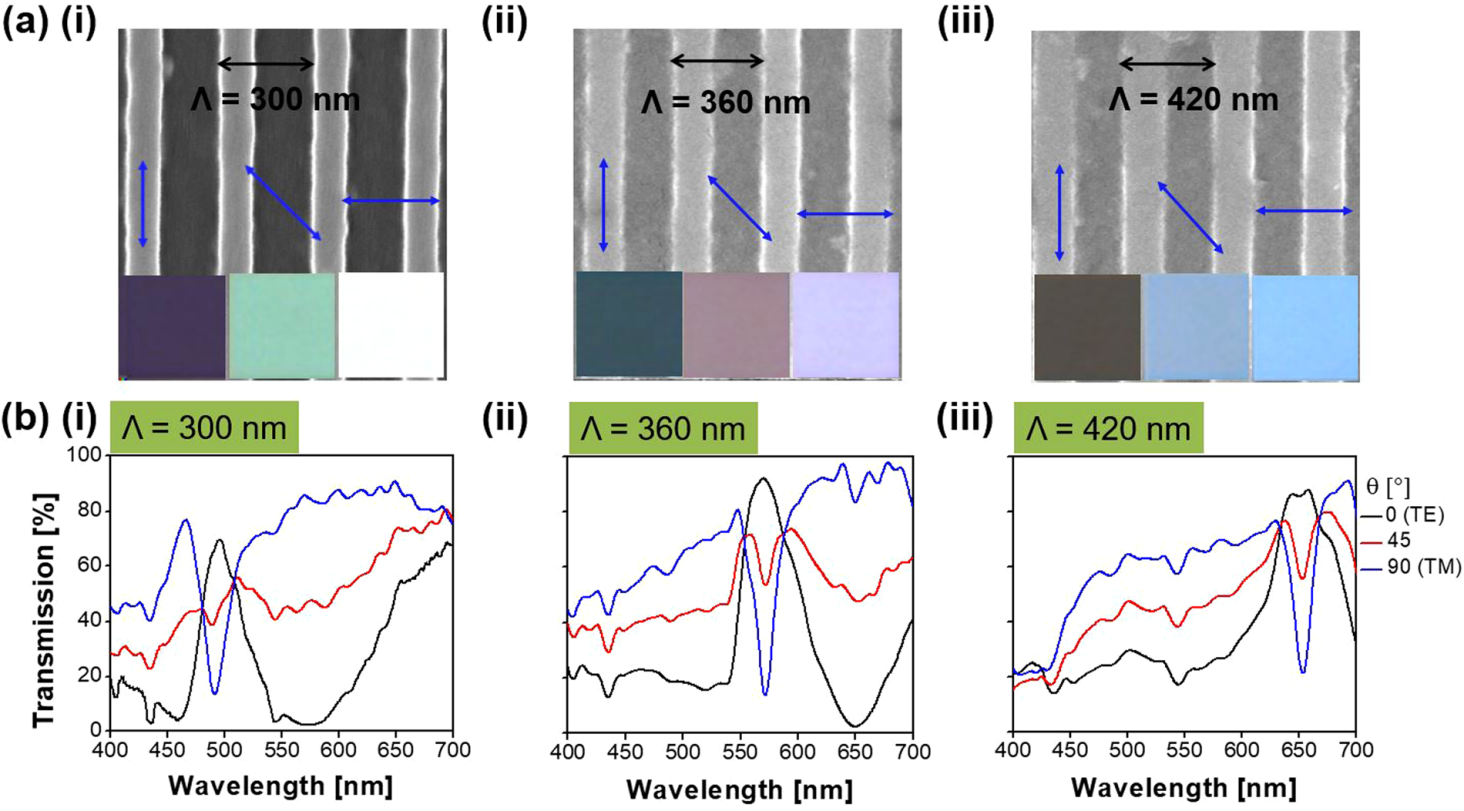
Fabricated MCPs and their spectral transmission. (**a**) SEM imagery, and (**b**) spectral responses in the transmission mode, depending on the polarization for the primary pixels (blue/yellow, green/magenta, and red/cyan) with periods of Λ = 300, 360, and 420 nm, respectively. The insets of (**a**) show the detected vivid color imagery for the TE and TM polarizations, in addition to the case of 45° polarization, indicated by a blue line, representing the electric field, with respect to the grating alignment.

Figure 1 shows that the proposed MCP consists of a 1D a-Si:H grating of 40-nm thickness (H_g_) that is formed on a silicon nitride core of 100-nm thickness (H_c_). Each pixel is of 40 µm × 40 µm dimensions. The duty ratio of the grating, defined as the ratio of the width (W) to the period (Λ), is chosen to be 0.35 to ensure enhanced transmissions for both the TE and TM polarizations. A single pixel gives rises to band-pass and band-stop filtering characteristics, which concur at the same spectral position. The polarization angle of incident light is indicated by the alignment of the electric (E) field with respect to the x-direction so that the TE and TM polarizations refer to the E-field that is aligned parallel (θ = 0°) and perpendicular (θ = 90°) to the grating, respectively. The proposed devices are deemed to work as transmissive spectral filters in the visible band by virtue of the GMR, which will be discussed later. For a specific polarization, the transmittance is expressed by the following relationship:

1 $${T}_{\theta }(\lambda )={T}_{0}(\lambda ){\cos }^{2}\theta +{T}_{90}(\lambda ){\sin }^{2}\theta $$

where *λ* is the free-space wavelength of the incident light, *θ* is the polarization angle, and *T*_0_ and *T*_90_ are the transmittance for the TE and TM cases, respectively. Figure 2(a) shows the SEM imagery of the fabricated MCPs tapping into an a-Si:H grating of varying pitches. The insets display the vivid color images taken under the microscope, depending on the polarization as indicated by the alignment of the blue arrow with respect to the grating. The polarization-sensitive transmission spectra are plotted in Fig. 2(b),(i) through (iii) in the cases of Λ = 300, 360, and 420 nm for θ = 0°, 45°, and 90°, respectively. The peak transmission for the prepared MCPs has been demonstrated to reach up to 92% for the TE polarization and 95% for the TM polarization. Three structural MCPs are designed to be centered at wavelengths of 493 (blue/yellow), 571 nm (green/magenta), and 653 nm (red/cyan), respectively, showing the band-pass and band-stop characteristics corresponding to the TE and TM cases. The difference between the measured resonant wavelengths for the TE and TM is found to be less than 5 nm. It is noted that, as shown in Supplementary Figure S1, the transmission peak efficiency has been calculated to be about 95% for the TE and for TM polarization, which are discovered to correlate decently with the demonstrated results, shown in Fig. 2(b).

### Generation of a suite of colors based on the polarization-controlled transmission spectra

**Figure 3:**
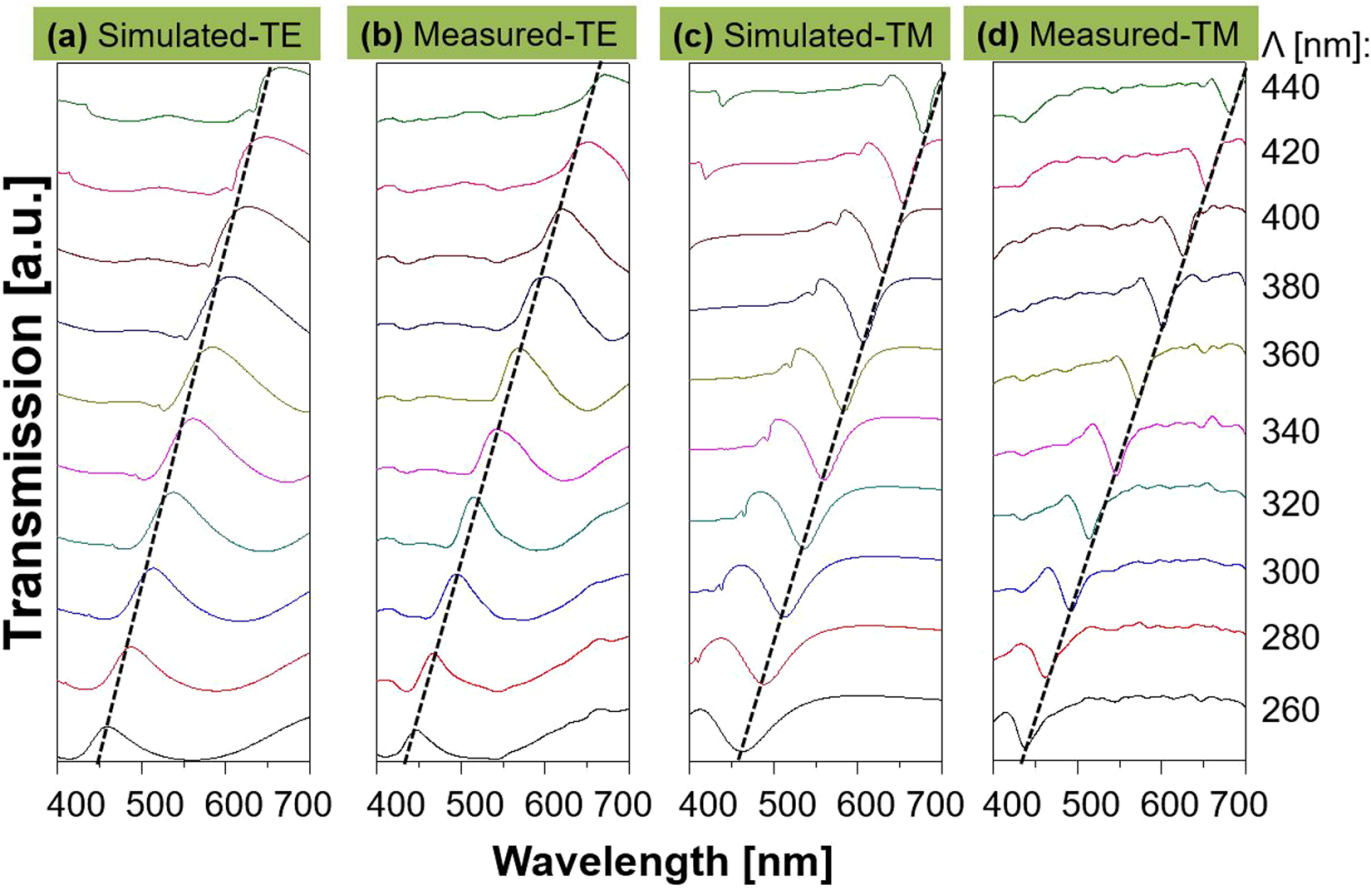
Polarization-dependent transmission with respect to the grating pitch. Simulated (**a**) and (**c**), and measured (**b**) and (**d**), transmission spectra for the TE and TM illuminated light, respectively, when the period is varied from 260 to 440 nm. The transmission peak and dip for the TE and TM, respectively, are traced by black dashed lines.

For the proposed device, the impact of the grating period on the spectral performance was rigorously investigated with the assistance of the finite difference time domain (FDTD) method. Figure 3 shows the simulated and measured transmission spectra when the period is scanned from 260 to 440 nm in increments of 20 nm. The transmission peak and dip correlate for the TE and TM polarizations. As indicated by the dashed black lines, the resonant wavelengths are traced to run from λ = 448 to 671 nm and λ = 438 to 681 nm in the calculation and measurement results for the TE and TM cases, respectively. The slight discrepancy between the calculated and measured efficiencies is thought to be ascribed to the surface roughness in conjunction with the errors during device fabrication. Meanwhile, the relatively large extinction coefficient of a-Si:H in the shorter wavelength region is held accountable for the degradation in transmission, indicating that the color purity of the images may be enhanced by suppressing the extinction coefficient of the material, particularly in the blue regime, through the optimization of its deposition process. The color purity for the images for the TM case is low relative to that for the TE, which is attributed to the fact that the TM transmission spectra showing a narrow bandwidth under the subtractive mode^25^. Supplementary Figure S2 shows that the bandwidth for the TM case increases with the a-Si:H thickness.

**Figure 4:**
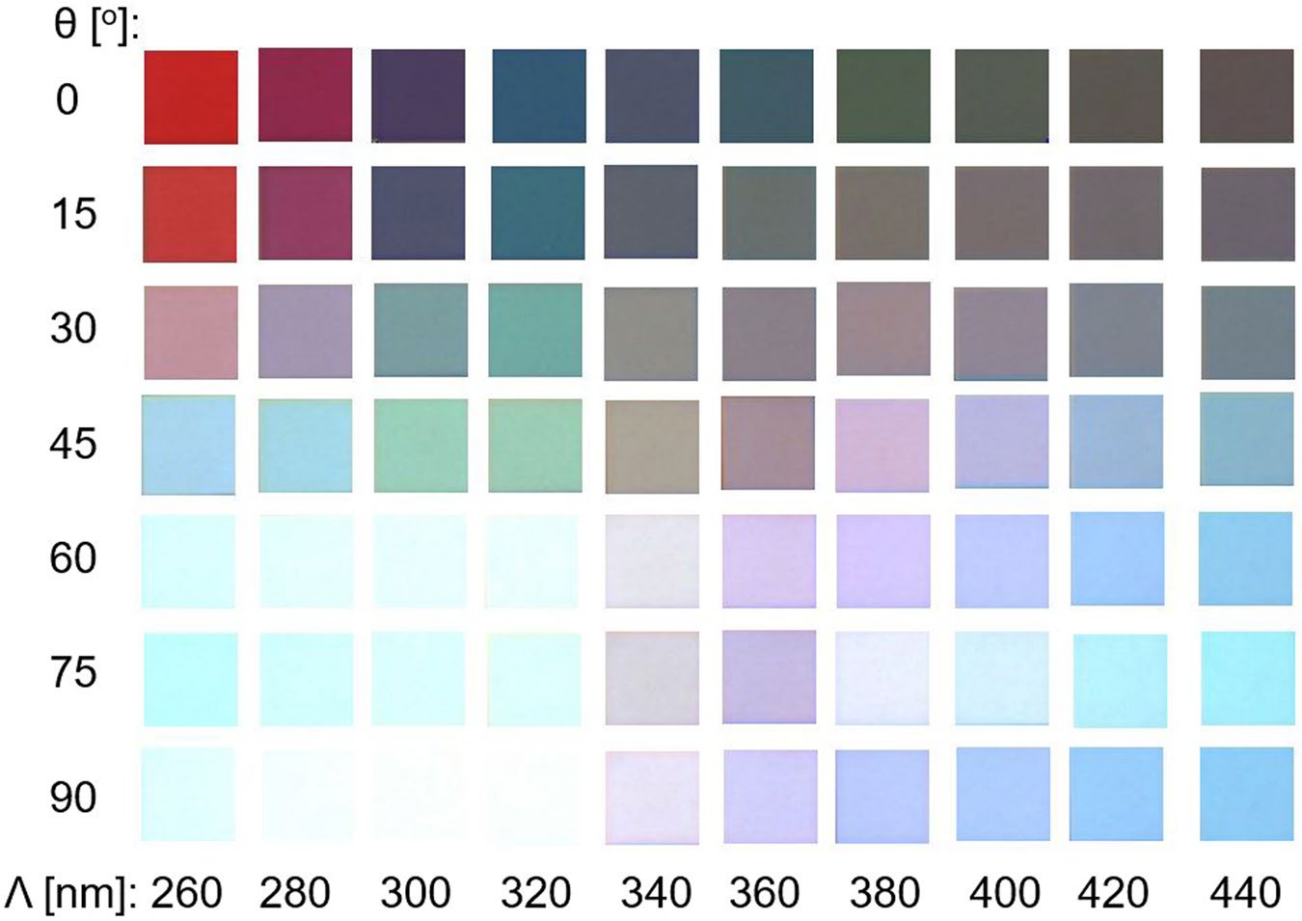
Bright-field microscope color imagery of the structural MCP in the transmission mode in terms of the grating period and the incident polarization. Each pixel has dimensions of 40 µm × 40 µm and the grating period is altered from 260 to 440 nm, while the polarization angle is scanned from 0° to 90°.

Figure 4 shows the bright-field microscope color imagery in the transmission mode for the manufactured pixel, with dimensions of 40 µm × 40 µm. The MCP with a constant period is presumed to produce polarization-mediated colors. The colored images are arranged in accordance with the period varying from 260 to 440 nm while the polarization is altered from 0° to 90°. A broad color palette can therefore be efficiently attained. The angular tolerance of the proposed MCP was then pursued in light of its practical applications. Supplementary Figure S3 shows the contour maps for the simulated transmission spectra in terms of the angle of incidence for the TE and TM cases for the pixel with a 340-nm pitch. The pixel is observed to provide an angular tolerance of ~35°, incurring no significant variations in the transmission.

### Mechanism for the polarization-dependent transmission peak and dip

**Figure 5:**
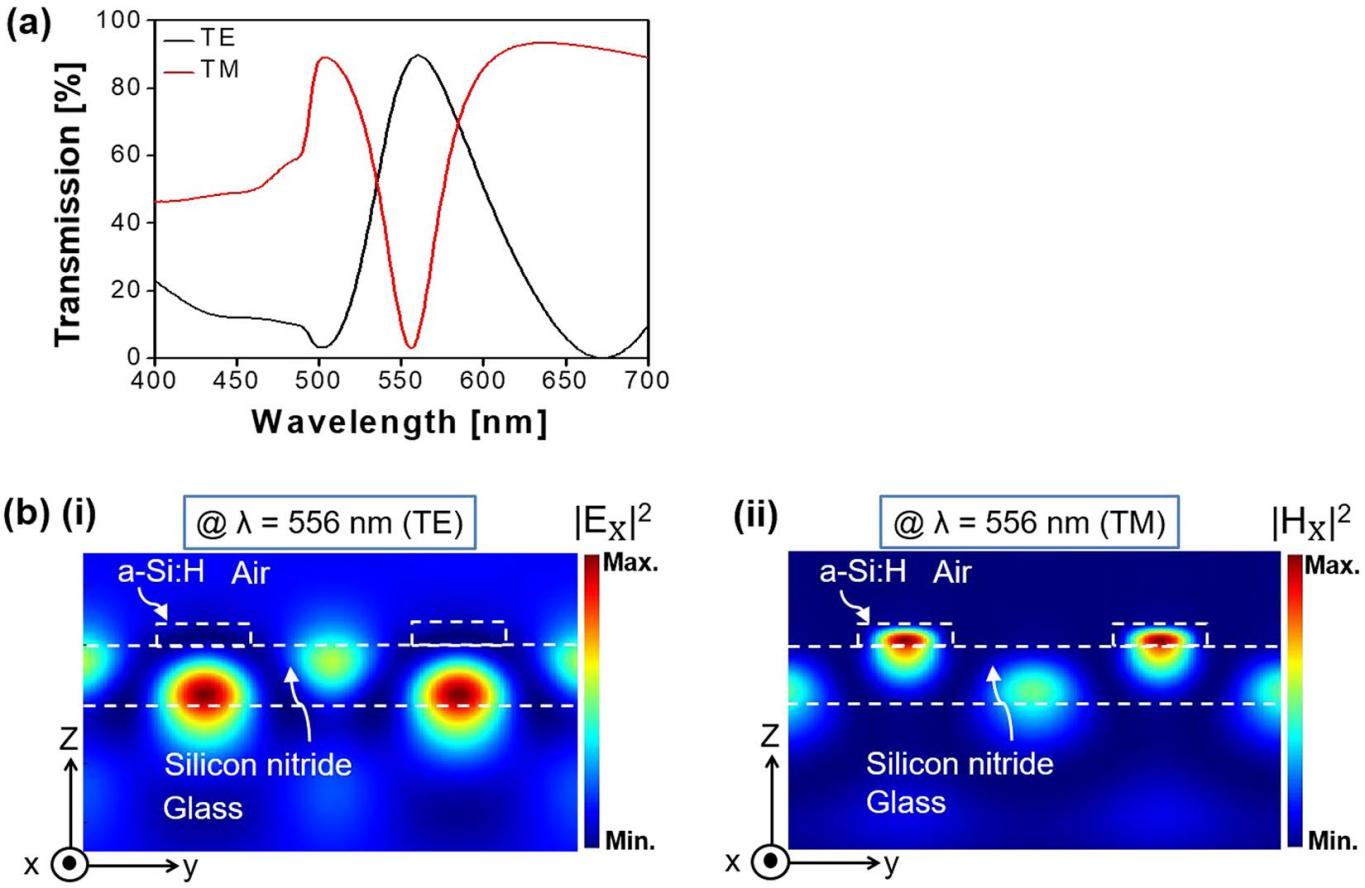
Calculated transmission spectra and field intensity profile for the MCP with Λ = 340 nm. (**a**) The resonant peak for the TE polarization and the resonant dip for the TM case. (**b**) Calculated (i) electric field intensity (|E_X_|^2^), and (ii) magnetic field intensity (|H_X_|^2^), observed at λ = 556 nm for the TE and TM cases, respectively.

**Figure 6:**
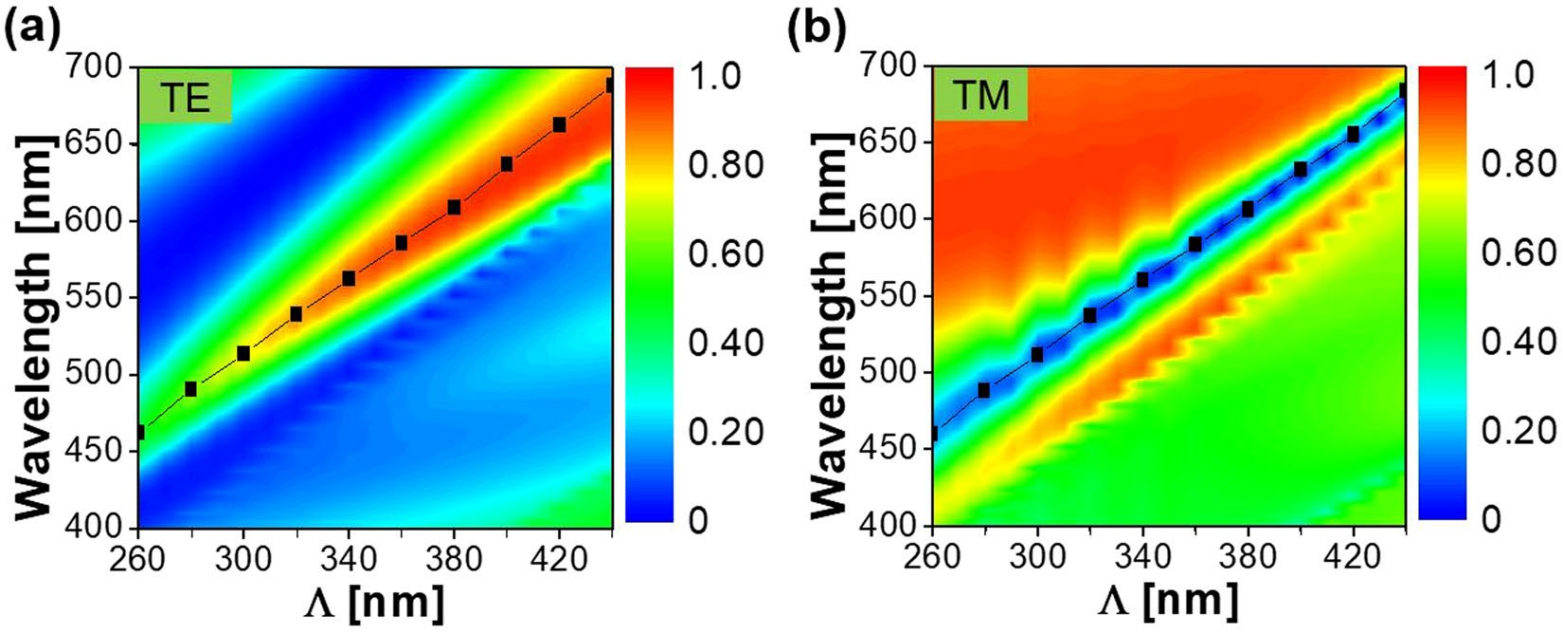
Verification of the GMR. The calculated GMR dispersion relation indicated by the black squares is superposed on the (**a**) TE, and (**b**) TM transmission spectra.

For the purpose of exploring the physical mechanism underpinning the polarization-dependent resonant peak and dip in the transmission, the electric- and magnetic-field intensity profiles were meticulously monitored for a typical pixel with Λ = 340 nm and a duty ratio of 0.35. Figure 5(a) shows the transmission spectra while Fig. 5(b) shows the E-field intensity (|E_x_|^2^) and the H-field intensity (|H_x_|^2^) for the TE and TM polarizations, respectively, corresponding to λ = 556 nm. It appears that |E_x_|^2^ for the TE and |H_x_|^2^ for the TM are simultaneously substantially reinforced within the core of the silicon nitride waveguide, corresponding to the transmission peak and dip. Supplementary Figure S4 shows that for the TM case, the transmission dip is related to the reflection peak, when the enhanced field in the dielectric core is slightly displaced towards the a-Si:H layer. The drastically strengthened field distributions signify the development of a standing wave that originates from the interaction of counter-propagating guided modes supported by the planar dielectric waveguide^26^. Figure 6(a) and (b) verify the presence of the GMR for the TE and TM polarizations, respectively, through the calculated dispersion and the contour map of the transmission^27^. The dispersion relation associated with the dielectric waveguide that is overlaid with the a-Si:H grating plays a prime role in deriving the GMR conditions, which relation is given by:

2 $$\begin{array}{ccc}m\pi  & = & {H}_{c}(\sqrt{{{k}_{0}}^{2}{{n}_{h}}^{2}-{\beta }^{2}})-ta{n}^{-1}({({n}_{h}/{n}_{c1})}^{2\rho }\sqrt{({\beta }^{2}-{{k}_{0}}^{2}{{n}_{c1}}^{2})/({{k}_{0}}^{2}{{n}_{h}}^{2}-{\beta }^{2}))}\\  &  & -ta{n}^{-1}({({n}_{h}/{n}_{c2})}^{2\rho }\sqrt{({\beta }^{2}-{{k}_{0}}^{2}{{n}_{c2}}^{2})/({{k}_{0}}^{2}{{n}_{h}}^{2}-{\beta }^{2}))}\end{array}$$

where, m is the mode number, H_c_ is the thickness of the core, β is the propagation constant, n_h_ is the refractive index of the core, n_c1_ is the effective index of the upper cladding which is relevant to the combination of the a-Si:H grating and air, n_c2_ is the refractive index of the substrate, and k_0_ is the wavenumber in free space.$\rho =$ 0 and 1 represent the TE and TM modes, respectively. The GMR is reckoned to take place when the phase matching is satisfied between the propagation constant β for the waveguide mode and the grating vector (G = 2π/Λ)^27^.

Supplementary Figures S2 and S5 show that the determinations of the thicknesses of a-Si:H and silicon nitride are scrutinized to elevate the transmission efficiency and relax the fabrication tolerance with the help of the transmission contour map. The efficiency was nearly insensitive to the thickness of the a-Si:H grating while the spectra red-shifted with the thickness of the dielectric core. The silicon nitride guiding layer integrated with an a-Si:H grating has been confirmed to facilitate the enhancement of color extinction. Supplementary Figure S6 shows the effect of the duty ratio on the transmission. The device with the duty ratio of 0.35 delivered the optimum performance in terms of the efficiency, the coincidence of the resonant wavelengths, and the acceptably small sideband. Moreover, according to the reflection characteristics presented in Supplementary Fig. S4, color tuning mediated by the polarization and the grating pitch is readily supported. Consequently, the proposed MCP is categorically presumed to accomplish a polarization-tailored spectral response in both the transmission and reflection modes.

## Discussion

Highly efficient polarization-mediated structural MCPs, capitalizing on an a-Si:H grating in conjunction with a dielectric waveguide, have been devised and developed, to span the primary cases of blue/yellow, green/magenta, and red/cyan. For the three manufactured MCPs, the transmission peak exceeds 70% for the TE polarization and 90% for the TM polarization as observed at the resonance and off-resonance wavelength, respectively. A broad color palette was obtained to cover the entire visible band through alteration of the grating pitch and dynamical control of the incident polarization. The TE transmission peak and the TM transmission dip, sharing the common resonant wavelength, were attributed to the GMR which is initiated by the grating integrated with the dielectric waveguide. For the bandpass and bandstop filtering characteristics, the near-field electric and magnetic intensity profiles, respectively, were inspected for the TE and TM incidence. The proposed devices are sure to lead to the emergence of many intriguing applications, including visible light communications, polarization-sensitive optical data storage, security tags, display devices, image sensors, and biomedical imaging devices.

## Methods

### Numerical simulations

The transmission spectra and the field profiles for the structural color pixels were explored with the assistance of an FDTD tool (FDTD Solutions, Lumerical, Canada)^28^. A plane wave under normal incidence was considered for the refractive indices of silicon nitride, a-Si:H, and SiO_2_^29^. A unit cell satisfying appropriate periodic boundary conditions was used to mimic the a-Si:H grating.

### Device fabrication

The proposed MCPs were designed and created to dimensions of 40 μm × 40 μm. A 100-nm-thick silicon nitride and 40-nm-thick a-Si:H film were successively deposited on a glass substrate using plasma enhanced chemical vapor deposition (PECVD) (Oxford, Plasmalab System 100 Dual Frequency). A 1D grating was subsequently patterned via an electron-beam lithography system (RAITH 150), for which a positive photoresist of ZEP520A was adopted. The a-Si:H film was finally etched using a plasma etcher (Oxford, Plasmalab System 100), under a gas mixture of CHF_3_ and SF_6_.

### Optical characterization

The completed a-Si:H pattern was visually inspected under high-resolution field emission scanning electron microscopy (FESEM S-4800, Hitachi). The transmission spectra were checked for different polarizations by launching a collimated beam available from a halogen lamp (HL-2000-FHSA, Ocean Optics), which was polarized through a calcite crystal polarizer (GTH 10M-A, Thorlabs), to the prepared pixel that was mounted on a motorized rotation stage. The optical output was captured by spectrometry (Avaspec-3648, Avantes) via a multimode fiber. The images for each color was captured via digital microscopy (Leica DM4000 M).

## Electronic supplementary material

Supplementary information
