# Supplementary material for: All Dielectric Transmissive Structural Multicolor Pixel Incorporating a Resonant Grating in Hydrogenated Amorphous Silicon
Source: Sci Rep. 2017 Oct 19;7:13574. doi: 10.1038/s41598-017-14093-6 (PMC5648771; doi:10.1038/s41598-017-14093-6)
Supplement: Supplementary file 1 — Supplementary information [file 41598_2017_14093_MOESM1_ESM.pdf]

SUPPLEMENTARY INFORMATION

All Dielectric Transmissive Structural Multicolor Pixel Incorporating a Resonant Grating in Hydrogenated Amorphous Silicon

Ishwor Koirala[1], Vivek Raj Shrestha[1,2], Chul-Soon Park[1], Song Gao[1], Sang-Shin Lee[1,*] and Duk-Yong Choi[3]

[1]Department of Electronic Engineering, Kwangwoon University, 20 Kwangwoon-ro, Nowon-gu, Seoul 01897, South Korea

[2]School of Physics, The University of Melbourne, Melbourne, Victoria 3010, Australia

[3]Laser Physics Centre, Research School of Physics and Engineering, Australian National University, Canberra ACT 2601, Australia

*Correspondence and requests for materials: slee@kw.ac.kr

Keywords: Nanoscale Devices, Displays, Optical Physics.

1. **Simulated transmission spectra for the polarization-sensitive structural color pixel with the grating period.**

The simulated transmission spectra for the MCP with Λ = 300, 360, and 420 nm, representing the primary color (blue/yellow, green/magenta, and red/cyan) bands, are shown below for different angles of polarization. The resonant wavelength is observed to be stably maintained regardless of the angle.

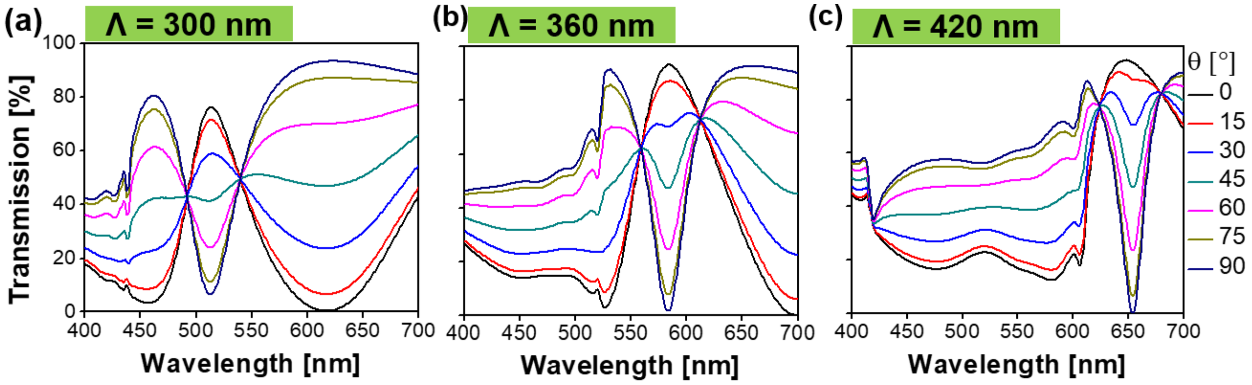

Supplementary Figure S1. Simulated transmission spectra of the pixels with grating pitches of (**a**) 300, (**b**) 360, and (**c**) 420 nm, for the polarization angle ranging from θ = 0 to 90°.

2. Device performance with the thickness of a-Si:H layer.

Supplementary Figure S2 explores the selection of the a-Si:H thickness (H_g) in relation to the enhanced transmission and flexible fabrication tolerance. The slight variations of H_g may be acceptable. By increasing H_g, the bandwidth of the transmission spectra decreases for the TE case, so as to degrade the color, while the bandwidth increases for the TM case, so as to improve the color purity.

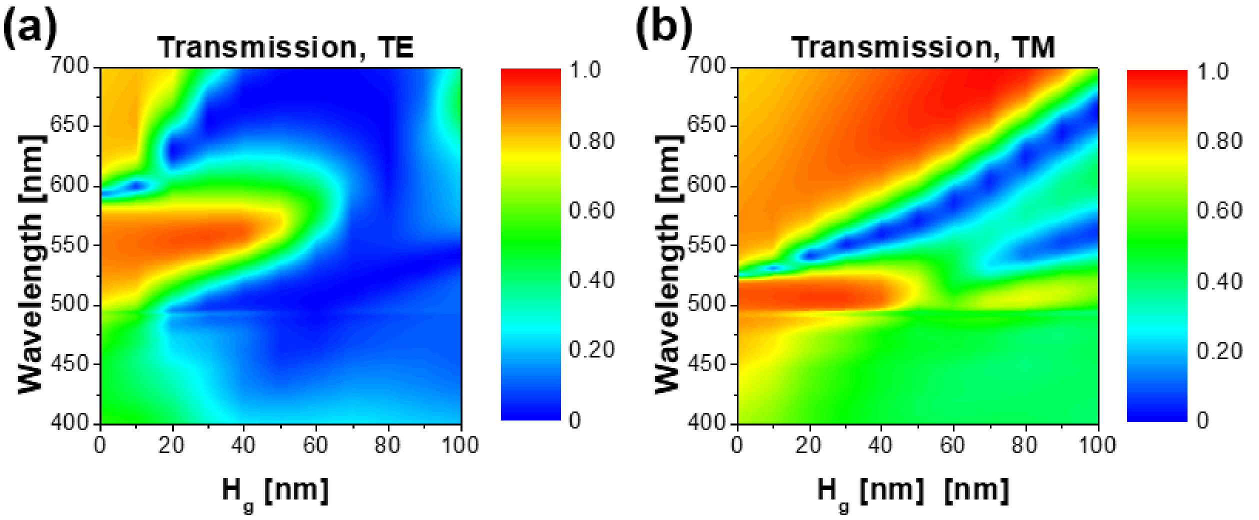

Supplementary Figure S2. Contour plot for the simulated transmission spectra for the grating period of 340 nm for (**a**) TE and (**b**) TM polarizations with the a-Si:H thickness varying from 0 to 100 nm.

3. Device performance in terms of the angular tolerance.

The angular performance for the pixel with a grating period of 340 nm was considered. Supplementary Figure S3 shows the contour map for the simulated transmission spectra for the angles of incidence up to 45° for the TE and TM cases. The pixel features an angular tolerance up to 35°.

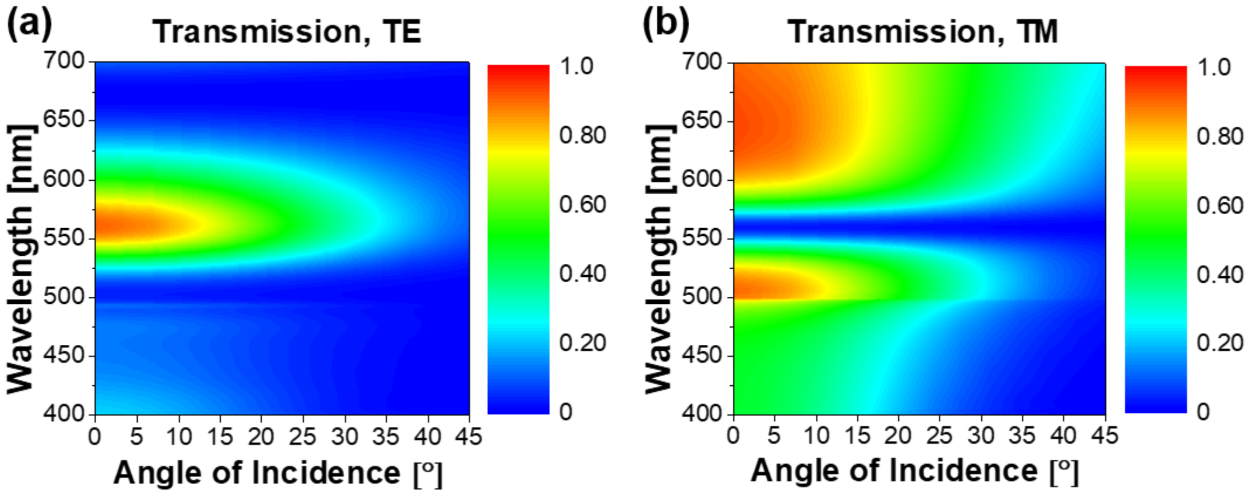

Supplementary Figure S3. Calculated angular tolerance for (a) TE, and (b) TM polarizations, for the case of Λ = 340 nm.

4. Reflection spectra of the proposed device.

Supplementary Figure S4 shows the calculated reflection spectra of the color pixel with the same structural parameters as those of the transmission case shown in Fig. 3. The spectral performance in the reflection mode implies similar color tuning.

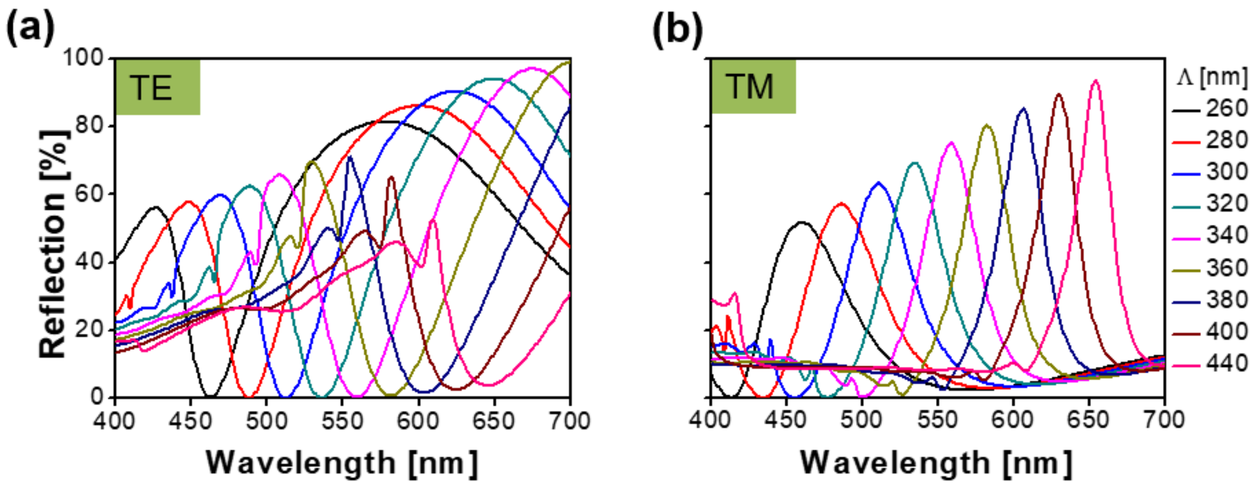

Supplementary Figure S4. Simulated reflection spectra when the grating period is altered from 260 to 340 nm for (**a**) TE, and (**b**) TM polarizations.

5. Device performance with the thickness of the silicon nitride layer.

Supplementary Figure S5 shows the transmission for the TE and TM polarizations for the silicon nitride film of 100-nm thickness. It is noted that the introduction of silicon nitride plays a pivotal role for the enhancing the color performance. The spectra are monitored to slight red shift, in response to the increase in thickness of the silicon nitride layer.

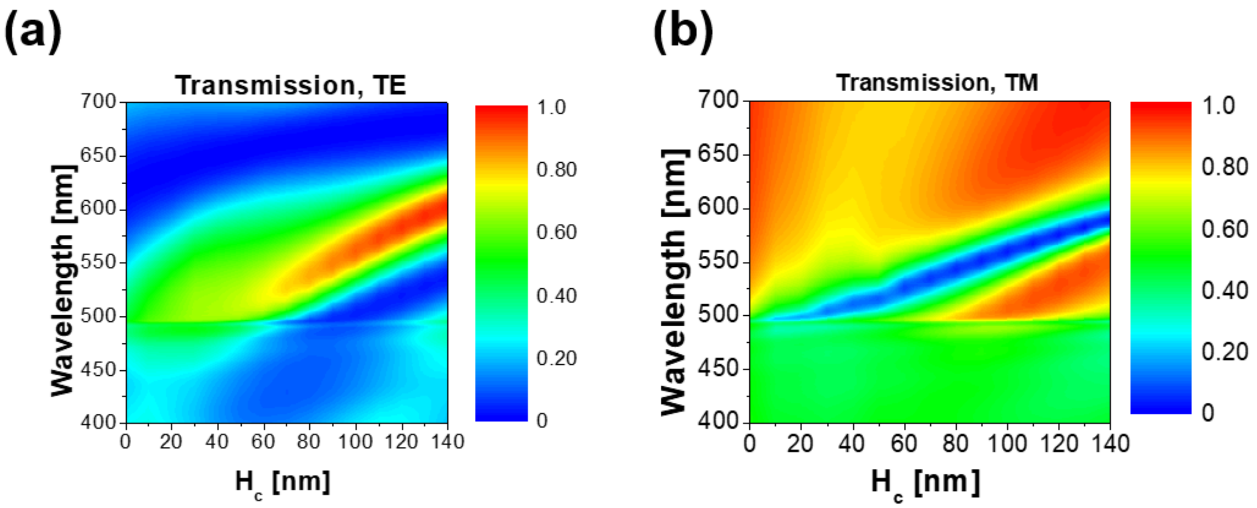

Supplementary Figure S5. Simulated transmission spectra for the pixel with a grating period of 340 nm for (**a**) TE, and (**b**) TM polarizations, with the thickness of the silicon nitride layer varying from 0 to 100 nm.

6. Device performance in terms of the duty ratio.

Supplementary Figure S6 shows the influence of the duty ratio on the transmission. The devices with a duty ratio of 0.35 are discovered to exhibit the best performance for the TE and TM polarizations, in terms of the transmission efficiency, coincident resonant wavelength, and sideband.

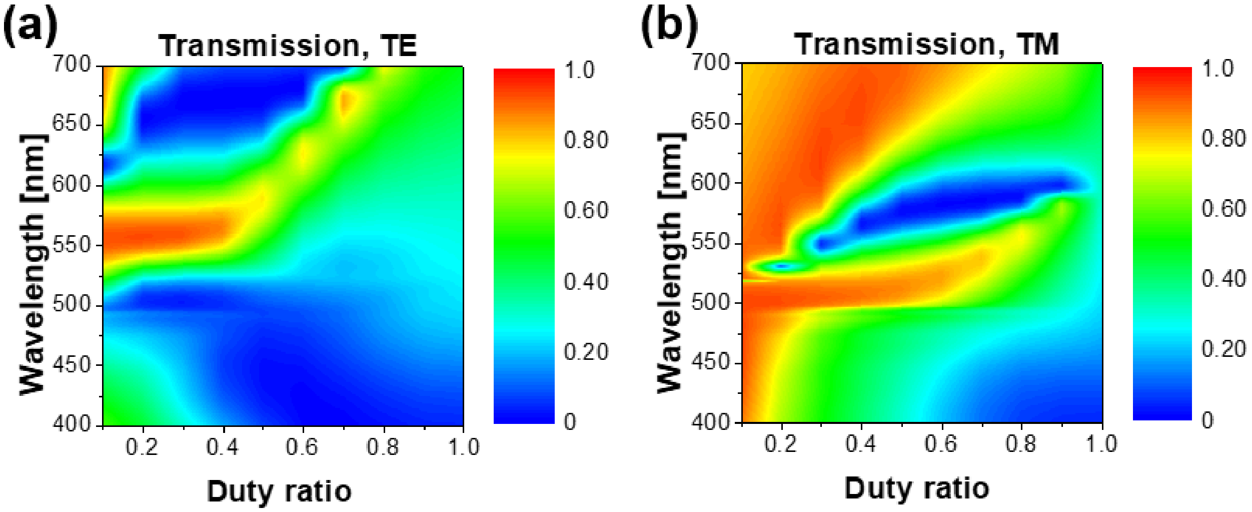

Supplementary Figure S6. Simulated transmission spectra for the pixel with a grating pitch of 340 nm for (**a**) TE, and (**b**) TM polarizations, when the duty ratio is changed from 0.1 to 1.
